# Therapeutic Clinical Trial Eligibility and Enrollment among Women with Breast Cancer: Implications for Understanding Trial Disparities

**DOI:** 10.1245/s10434-024-16607-9

**Published:** 2024-12-09

**Authors:** Nicole Reh, Nicole E. Caston, Courtney P. Williams, Sindhu R. Dwarampudi, Ahmed Elkhanany, Katia Khoury, Erica Stringer-Reasor, Nusrat Jahan, Gabrielle B. Rocque, Lily A. Gutnik

**Affiliations:** 1 The University of Alabama at Birmingham Heersink School of Medicine, Birmingham, AL USA; 2 Cancer Care Quality Training Program, UNC Lineberger, Chapel Hill, NC USA; 3 General Internal Med and Population Science, The University of Alabama at Birmingham Heersink School of Medicine, Birmingham, AL USA; 4 Medical College of Georgia, Augusta, GA USA; 5 Medicine- Hematology and Oncology, Baylor College of Medicine, Houston, TX USA; 6 The University of Alabama at Birmingham Heersink School of Medicine, Medicine-Hematology and Oncology, Birmingham, AL USA; 7 The University of Alabama at Birmingham Heersink School of Medicine, Surgery, Birmingham, AL USA

## Abstract

**Introduction:**

Therapeutic clinical trials frequently lack diverse representation, hindering generalizability and exacerbating preexisting disparities in clinical outcomes. This study explored associations between breast cancer patient demographics, clinical trial eligibility, and enrollment in a National Cancer Institute (NCI)-designated cancer center.

**Patients and methods:**

This prospective cohort study included patients with breast cancer screened for therapeutic clinical trials from July 2020 to January 2024. Eligibility was determined by the provider and study coordinator. Patient characteristics were abstracted from the electronic medical record. Rurality and neighborhood disadvantage were mapped by address using rural–urban commuting area codes and area deprivation index (ADI), respectively. Likelihood of eligibility and enrollment by race, rurality, and neighborhood disadvantage were evaluated using risk ratios (RR) and 95% confidence intervals (CIs) from modified Poisson regression models.

**Results:**

Of 343 patients screened for therapeutic trials, the mean age was 56 years (SD 13), 33% were Black/other race, 22% lived in highly disadvantaged areas, and 16% in rural areas. Most patients were screened for one trial (87%). Overall, 54% of patients were eligible for trials, and of those, 58% enrolled. Similar likelihoods of eligibility and enrollment were seen by race and rurality. Though not significant, patients living in highly disadvantaged areas trended toward higher likelihood of enrollment (RR 1.24, 95% CI 0.99–1.55).

**Conclusions:**

Over half of trial-eligible patients, even across race, rurality, or neighborhood disadvantage, enrolled, surpassing the national average. In contrast to national trends, there was higher enrollment among patients of higher ADI.

Clinical trials have led to the development and dissemination of new findings, screenings, treatments, and other advancements in the landscape of breast cancer.^1^ However, patients participating in cancer clinical trials are often unrepresentative of the real-world patient populations. Nationally, only a fraction of cancer patients qualify for clinical trial participation, with enrollment rates ranging from 4 to 20% for all patients, and 50% for those eligible.^2–4^ There are multiple junctures in the participation cascade that could systematically exclude patients from participating. The cascade to participation is as follows: eligibility is determined, interest is investigated, then enrollment onto the trial occurs. If a patient is eligible, participation is further influenced by many factors. Common barriers cited in the decision to participate in a trial include patient mistrust in the provider or health system, a lack of a patient-centered approach, poor patient-provider relationships, cost, transportation, lack of clinical trial knowledge, time commitments, fear, and family issues.^5,6^ A greater understanding of these barriers is needed, especially for patients potentially eligible for cancer clinical trials in the Deep South.

Though information gleaned from these trials depends on adequate and representative patient participation, diversity in breast cancer clinical trials is also lacking. Over 80% of therapeutic agents derived from clinical trials are based on in vitro models and cell lines predominantly sourced from women of European ancestry.^7^ Furthermore, patients from racial and ethnic minoritized groups, rural areas, and socioeconomically disadvantaged areas are consistently underrepresented in clinical trials.^8–10^ Enrollment of Black patients is 11-fold lower than white patients;^4^ patients of low socioeconomic status are 27% less likely to participate compared with those of higher socioeconomic status,^11^ and patients from rural areas are half as likely to enroll than urban counterparts.^4^ This lack of diversity in trial populations undermines the applicability and generalizability of trial results to broader patient populations.

To fully grasp and address disparities among participation in breast cancer clinical trials, we must consider the unique barriers facing different patient populations at various steps of the pathway towards clinical trial participation. This study seeks to describe and explore eligibility and enrollment patterns for breast cancer therapeutic clinical trials at a National Cancer Institute- (NCI)-designated comprehensive cancer center located in the Southeast, particularly evaluating the impact of race, socioeconomic factors, and urban/rural patient location.

## Methods

### Study Design and Participants

Medical oncologists along with research coordinators at our institution maintain an internal database of breast cancer patients who were seen and assessed for their eligibility for potential trials and subsequently tracked their enrollment. This database was used to retrospectively analyze eligibility and enrollment among patients with breast cancer who were screened for therapeutic clinical trials at the University of Alabama at Birmingham (UAB) from July 2020 through January 2024. For this analysis, inclusion criteria were: stage I–IV breast cancer, female sex due to low frequency of male patients in our dataset, complete address information, complete race information, complete marital status information, and patients who were referred for clinical trial screening at least once. Patients who were referred for a clinical trial and did not appear or cancelled their appointment were excluded. This study was approved by the University of Alabama at Birmingham Institutional Review Board (300001910).

### Outcomes: Clinical Trial Eligibility and Enrollment

*Eligible for a Clinical Trial:* Eligibility for a clinical trial was determined by the provider and research coordinator for trials open and available at UAB. Eligibility was reported as a yes or no.

*Enrollment onto a Clinical Trial: *Patient enrollment status was documented for trials open and available at UAB. Enrollment was reported as a yes (agree) or no (decline).

### Exposures: Patient Race, Rurality, and Neighborhood Deprivation

Data on patient race and address were abstracted from the electronic health record (EHR).

*Race*: Race (Black, white, Asian, American Indian, or Alaskan) was self-reported by the patient. Race was analyzed as white or Black/Other. Other included those who self-reported as Asian, American Indian, or Alaskan Native.

*Rurality*: Patient home address zip code was used to determine rurality using rural–urban community area codes (RUCA). RUCA codes utilize census tract-based classifications, including standardized measures of population density, levels of urbanization, and work commutes, to block group 12-digit federal information processing standard publication codes to rural or urban designations.^12^

*Neighborhood Disadvantage*: Neighborhood disadvantage, a proxy for socioeconomic status, was classified as either low neighborhood disadvantage or higher neighborhood disadvantage. Area deprivation index (ADI) measures were calculated using patient address and scored in percentiles ranging from 1% to 100%. An ADI measurement of 1% indicates the lowest level of disadvantage, and 100% indicates the highest level of disadvantage. Lower disadvantaged areas are defined by ADI measurements of 1–85%, while higher disadvantaged areas include the upper 15th percentiles (85–100%).^13^

### Patient Demographics and Clinical Characteristics

Patient age at the time of trial referral, self-reported sex, breast cancer staging and information, comorbidity status (via ICD–10 codes), and marital status were abstracted from the EHR. The Elixhauser comorbidity index was utilized to categorize patient comorbidities by international classification of diseases, 10th edition (ICD–10) diagnosis codes abstracted from patient EHR.^14^

### Statistical Analyses

Descriptive statistics were calculated using frequencies and percentages for categorical variables, further utilized for crude eligibility and enrollment analyses. Descriptive statistics were also calculated using means and standard deviations (SD) for continuous variables. Crude rates of eligibility and enrollment were compared using chi-squared tests. Risk ratios (RR) and 95% confidence intervals (CI) were estimated from two modified Poisson regression models with robust error variance. The first model estimated the likelihood of trial eligibility by patient race, rurality, and neighborhood disadvantage. The second model estimated the likelihood of trial enrollment by patient race, rurality, and neighborhood disadvantage. Both models were controlled for age at trial referral, marital status, cancer stage, tumor biology, and comorbidities. Analyses were performed using SAS software, version 9.4 (SAS Institute, Cary, NC).

## Results

### Sample Characteristics

**Fig. 1:**
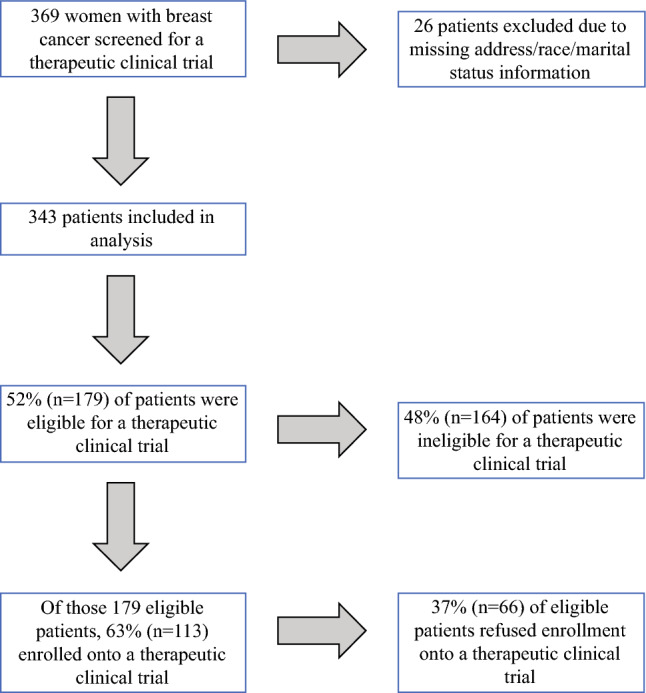
Clinical trial eligibility and enrollment among breast cancer patients at an academic NCI-designated cancer center

Overall, 343 out of 369 patients screened at least once for a breast cancer clinical trial were included in our study (*n* = 400 screenings total). Patient characteristics of those included were similar to those excluded (Table 1). The mean age of screened patients was 56 (SD 12.7) years, 33% were Black or other race, 22% lived in a disadvantaged neighborhood, and 16% lived in rural areas (Table 1). Most patients were screened for one trial (87%), while 13% were screened for multiple trials. Figure 1 demonstrates the cascade to clinical trial participation including patients that were excluded due to missing information. Of all patients screened, 52% were eligible for a trial. Of those eligible patients, 63% decided to enroll in a trial. Total enrollment among all patients screened was 33%.

**Table 1:** Patient characteristics (n = 343)

	*n* (%)
Average age at trial referral (Mean, SD)	56 (SD = 12.7)
*Neighborhood disadvantage*	
Less disadvantage	267 (78)
Higher disadvantage	76 (22)
*Residence type*	
Rural	55 (16)
Urban	288 (84)
*Race*	
White	229 (67)
Black/other	114 (33)
*Marital status*	
Single	159 (46)
Married	184 (54)
*Cancer stage*	
0	32 (9)
1	45 (13)
2	119 (35)
3	47 (14)
4	100 (29)
Tumor biology*	
HR+/HER2−	142 (41)
HR+/HER2+	39 (11)
HR−/HER2+	19 (6)
Triple negative	86 (25)
*Number of trials screened for*	
1	298 (87)
2	38 (11)
3 +	7 (2)
*Elixhauser comorbidity score*	
0	15 (4)
1–2	180 (52)
3+	125 (36)

*HR = Hormone Receptors (estrogen receptor and progesterone receptor), HER2 = human epidermal growth factor receptor 2, Triple negative = estrogen, progesterone, and human epidermal growth factor receptor 2 receptor negative

### Crude Eligibility and Enrollment Patterns

**Fig. 2:**
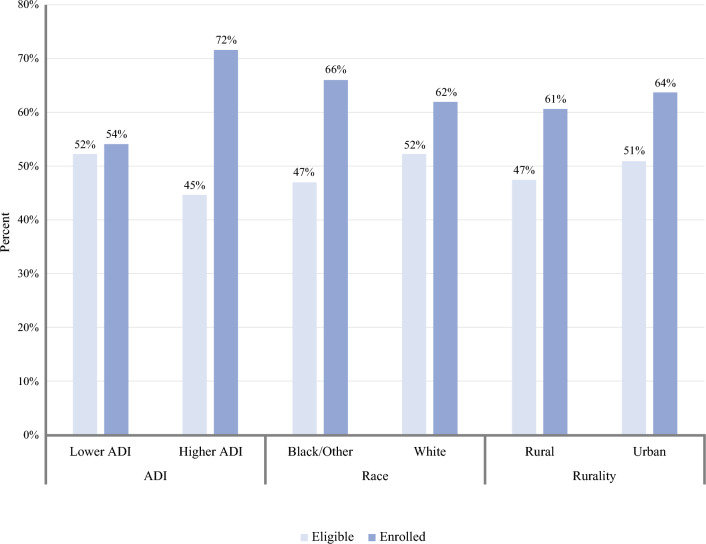
Comparison of unadjusted eligibility and enrollment rates by neighborhood disadvantage, race, and rurality (*n* = 400*). ^*^Patients could be screened/have eligibility assessed for more than one trial

Figure 2 demonstrates a comparison of patients eligible and enrolled by race, rurality, and neighborhood disadvantage. Similar unadjusted percentages of eligibility and enrollment were found between race and rurality. Black patients had similar crude rates of eligibility (47% versus 52%, *p* = 0.38) and enrollment (66% versus 62%, *p* = 0.68) compared with white patients. Similar unadjusted percentages of eligibility (47% versus 51%, *p* = 0.72) and enrollment (61% versus 64%, *p* = 0.92) were seen among rural and urban patients. Patients with higher neighborhood disadvantage had similar crude rates of eligibility (45% versus 52%, *p* = 0.24) compared with patients with lower neighborhood disadvantage. However, patients with higher neighborhood disadvantage had higher rates of enrollment (72% versus 54%, *p* = 0.047) compared with those with lower neighborhood disadvantage.

### Clinical Trial Eligibility

In adjusted models, Black and white patients had similar likelihoods of trial eligibility (RR 0.88, 95% CI 0.68–1.15), as did rural and urban patients (RR 0.97, 95% CI 0.73–1.30). There was no statistically significant difference in likelihood of eligibility between patients from higher and lower neighborhood disadvantage (RR 0.88, 95% CI 0.67–1.16; Table 2).

**Table 2:** Modified Poisson regression of therapeutic trial eligibility and enrollment risk ratios

	Risk Ratios (95%CI) estimating trial eligiblity	Risk Ratios (95%CI) estimating trial enrollment
	*N* = 400*	*N* = 225
Higher vs lower disadvantaged areas	0.88 (0.67–1.16)	1.24 (0.99–1.55)
Rural vs urban	0.97 (0.73–1.30)	1.04 (0.75–1.43)
Black/Other vs White	0.88 (0.68–1.15)	0.94 (0.72–1.19)

Model controlled for age at trial referral, marital support, cancer stage, tumor biology, and number of comorbidities according to the Elixhauser

Comorbidity Index. *Patients could be screened/have eligibility assessed for more than one trial*.*

### Clinical Trial Enrollment

In adjusted models, neither race, rurality, nor neighborhood disadvantage were independently associated with trial enrollment (Table 2). Black and white patients had similar likelihoods of trial enrollment (RR 0.94, 95% CI 0.72–1.19), as did rural and urban patients (RR 1.04, 95% CI 0.75–1.43). Area disadvantage, while not statically significantly, trended towards increased likelihoods of trial enrollment (RR 1.24, 95% CI 0.99–1.55).

## Discussion

In this study of women with breast cancer screened for a therapeutic breast cancer clinical trial at an NCI-designated cancer center in the Deep South, we found that more than half were eligible for therapeutic clinical trials. Furthermore, more than 50% of eligible patients, regardless of race, rurality, and area disadvantage, enrolled into a therapeutic clinical trial. Interestingly, there was no statistically significant difference between area disadvantage and eligibility for a clinical trial. Additionally, both crude rates of enrollment and univariate analyses demonstrated an association between higher area disadvantage and increased enrollment. While multivariable analyses did not demonstrate significance, a trend of increased likelihood of enrollment was observed. Our finding contrasts with existing literature associating lower socioeconomic status with decreased enrollment in trials.^15–17^ Financial factors and incentives could explain this, as economically vulnerable patients most commonly cite cost, including travel, missed work, and childcare, as a barrier to enrollment.^18,19^ While compensation and monetary incentives can enhance enrollment and accrual,^20–22^ further research is indicated to understand what specific patient-level factors are influencing participation in low income populations. Though, our research suggests that eligibility and patient acceptance of the trial may not be the primary drivers of lack of participation. It is well documented that patients from areas of higher disadvantage typically have both lower eligibility and enrollment compared with those from areas of lower disadvantage.^16^ This phenomenon is attributed to factors including limited access to healthcare, higher prevalence of multimorbidity, and later-stage presentation, all of which could impact the likelihood of patient eligibility.^20,23^ Such findings suggest that efforts to improve trial access and expand eligibility criteria may aid in including more socioeconomically diverse patients in cancer clinical trials.

We found no significant differences in trial eligibility or enrollment based on race. Furthermore, both enrollment and eligibility among Black patients at our institution was higher than the national average.^10^ This is particularly notable given that medical mistrust among Black patients remains significant in Alabama, due to the enduring impact of historical exploitative medical practices such as the 1932–1972 Tuskegee Syphilis study.^24^ Furthermore, recent literature highlights that ongoing perceived or real racism and discriminatory practices in healthcare and research exacerbate mistrust in Black patients today.^24,25^ Implicit bias among all medical personnel is an important consideration in the participation of minority patients, especially at our institution where 43.5% of the community we serve is Black. Specifically, physician implicit bias has been implicated as a barrier to representation of minority patients in trials, as it leads to inconsistent trial offers.^26^ To address this, the American Society of Clinical Oncology (ASCO) and the Association of Community Cancer Centers (ACCC) recommend internal evaluation to assess for racial and ethnic disparities in screening and referral, allowing for individualized adjustments to enhance diversity.^27^ Also, the ASCO and ACCC have implemented Just ASK*,* a training program designed to reduce physicians’ implicit bias by encouraging providers to ask all patients about trial participation. The training displayed successful results,^27^ and while our institution has not utilized this, it could be beneficial to increase eligibility and enrollment among all patients.

In our center, the first step to enrolling a patient on a clinical trial requires referral from a medical, surgical, or radiation oncologist. For this critical first step to occur, the physician must actively recall and identify an available trial, be familiar with the general inclusion criteria, and overcome any implicit bias. Further challenges that physicians report include time constraints, limited awareness of trials, and noncooperation from colleagues.^28^ Following physician referral in our center, a study coordinator takes over screening for eligibility based on specific criteria outlined in study protocols, including: laboratory values, pathologic characterizations, and comorbidity status.^29^ Generally, at this point, many patients are deemed ineligible due to the high prevalence of comorbidities and the extremely specific nature of trial criteria.^29^ Dedicated research staff are essential for this process, as providers alone may not have the capacity to manage the identification, enrollment, and follow-up.^30^

Our institution is fortunate to have clinical trial coordinators ultimately alleviating much of the physician burden which may account for why we did not see any significant differences in trial eligibility among various demographic groups. However, the process of screening and recruitment at our institution has room for refining and improvement. At the time of this study, mainly medical oncologists were often responsible for initiating the screening and recruiting process. They identified potential trials for their patients based on their main eligibility criteria (i.e., cancer subtype and stage), and either discussed it with the patient or referred the case to the trial coordinator for further recruitment and screening based on full trial eligibility. A database is maintained by the clinicians and research coordinators track each patient considered for a clinical trial to monitor their eligibility, consent, trial initiation, and trial termination. However, as of recently, screening for clinical trials is incorporated into our weekly multidisciplinary treatment planning meetings, where each new patient gets discussed, imaging reviewed, and preliminary plans made. This ensures that each patient is at least initially considered for a trial based on cancer type and stage, allowing for more intentional consideration of trial enrollment and ensuring that eligible patients are identified early in their treatment planning.

Similarly, we observed no significant disparities in trial enrollment based on rurality, with patients from both urban and rural areas demonstrating similar likelihoods of trial eligibility and enrollment. This finding contradicts previous literature, which cites lower trial participation rates among rural populations.^31^ Existing literature attributes this to factors including lack of facilities, inadequate infrastructure, inability to travel, lack of specialists, and financial barriers.^32^ Our findings could stem from the substantial focus on circumventing barriers to clinical trials for rural patients at our NCI-designated CCC in the Deep South. Such patients are demonstrating significant health seeking behaviors through pursuing our tertiary care. Also, many community physicians refer complex cancer patients to our center, sometimes for discussion of clinical trials when current standard of care options may be lacking or ineffective, further diversifying our patient population. While our institution has utilized cross-institutional collaborations, there remains opportunity to increase population-reflective accrual further through a more formalized community clinical trial network.^33^

Our study has several limitations. Our sample was drawn from a single institution located in the Deep South, which may limit the generalizability. Our database was prospectively internally maintained voluntarily by medical oncologists and study coordinators. The database was not exclusively designed for research purposes, thus may have missing or incorrect data entry. Finally, we focused exclusively on patients referred for therapeutic breast cancer trials, and findings may not generalize to other types of clinical trials or cancer populations.

## Conclusion

This study highlighted impressive rates of eligibility and enrollment for breast cancer clinical trials. Interestingly, race and rurality did not seem to influence eligibility or enrollment. However, socioeconomic factors may play a role in the decision to enroll in breast cancer clinical trials, and additional data is needed to determine their impact conclusively. Future research should explore strategies to address barriers to trial participation, particularly among underserved populations, to ensure that all patients can benefit from advances in cancer treatment.
